# Supplementary material for: Neuronal selectivity for stimulus information determines prefrontal LFP gamma power regardless of task execution
Source: Commun Biol. 2023 May 11;6:505. doi: 10.1038/s42003-023-04855-6 (PMC10175284; doi:10.1038/s42003-023-04855-6)
Supplement: Supplementary file 2 — Supplementary Material [file 42003_2023_4855_MOESM2_ESM.pdf]

Supplementary Material for:

Neuronal selectivity for stimulus information determines prefrontal LFP gamma power regardless of task execution

Balbir Singh[1], Zhengyang Wang[2], and Christos Constantinidis[1,2,3*]

1. Department of Biomedical Engineering, Vanderbilt University, Nashville TN 37235, USA.
2. Neuroscience Program, Vanderbilt University, Nashville TN 37235, USA.
3. Department of Ophthalmology and Visual Sciences, Vanderbilt University Medical Center, Nashville TN 37232, USA.

*Lead Contact:
Christos Constantinidis, Ph.D.
Department of Biomedical Engineering
Vanderbilt University
E-mail: Christos.Constantinidis.1@vanderbilt.edu

This PDF file includes:
Supplementary Table 1-2
Supplementary Figure 1-9

	AD	PD	MD	AV	PV	Total		AD	PD	MD	AV	PV	Total
Neurons in Spatial Set							Electrodes in Spatial Set						
Pre	206	84	441	74	269	1074	Pre	69	35	147	25	104	380
Post	118	181	361	102	417	1179	Post	56	66	154	52	172	500
Neurons in Shape Set							Electrodes in Shape Set						
Pre	36	79	283	3	238	639	Pre	13	32	91	2	86	224
Post	4	175	339	0	349	867	Post	3	61	135	0	152	351

Supplementary Table 1: Number of neurons and electrodes and recorded prior to training and after training, with the spatial and shape stimulus set. AD: Anterior-Dorsal; PD: Posterior-Dorsal; MD: Mid-Dorsal; AV: Anterior-Ventral; PV: Posterior-Ventral.

Monkey	Pre-Training			Post-Training		
	Recording Duration	Sessions	Trials	Recording Duration	Sessions	Trials
ADR	1 month	Spatial (16), Shape (17)	Spatial (7779), Shape (7074)	1 year, 2 months	Spatial (38), Shape (25)	Spatial (9770), Shape (5474)
ELV	3 months	Spatial (63), Shape (60)	Spatial (26437), Shape (23026)	1 year, 8 months	Spatial (117), Shape (116)	Spatial (38049), Shape (32692)
NIN	6 months	Spatial (59)	Spatial (22825)	10 months	Spatial (59)	Spatial (16987)

Supplementary Table 2: Number of neurons and electrodes and recorded prior to training and after training, with the spatial and shape stimulus set. AD: Anterior-Dorsal; PD: Posterior-Dorsal; MD: Mid-Dorsal; AV: Anterior-Ventral; PV: Posterior-Ventral.

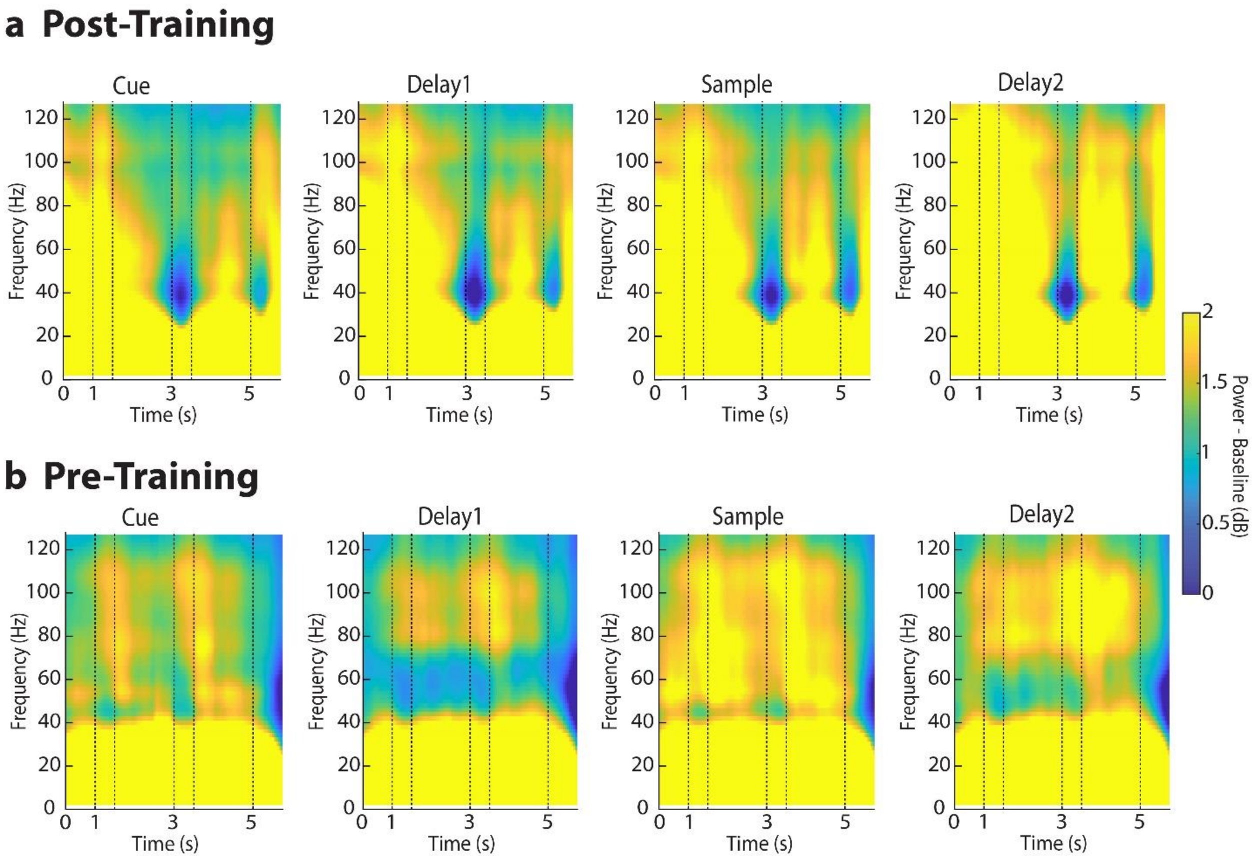

Supplementary Fig. 1: Power spectrum of sites with selectivity at different task intervals. LFP induced spectral power recorded with the spatial stimulus set from the prefrontal cortex, after training (**a**) and prior to training (**b**). In both cases, spectral power from selective sites is shown. Power is plotted as a function of time, after subtracting the mean power computed in the inter-trial interval at each frequency. **a** Trials from selective sites after training: n=9831 trials for the cue period, 6549 trials for the first delay period, 10440 trials for the sample period, and 6646 trials for the second delay period from selective sites. **b** Trials from selective sites prior to training: n=6091, 2726, 5201 and 2437 trials respectively.

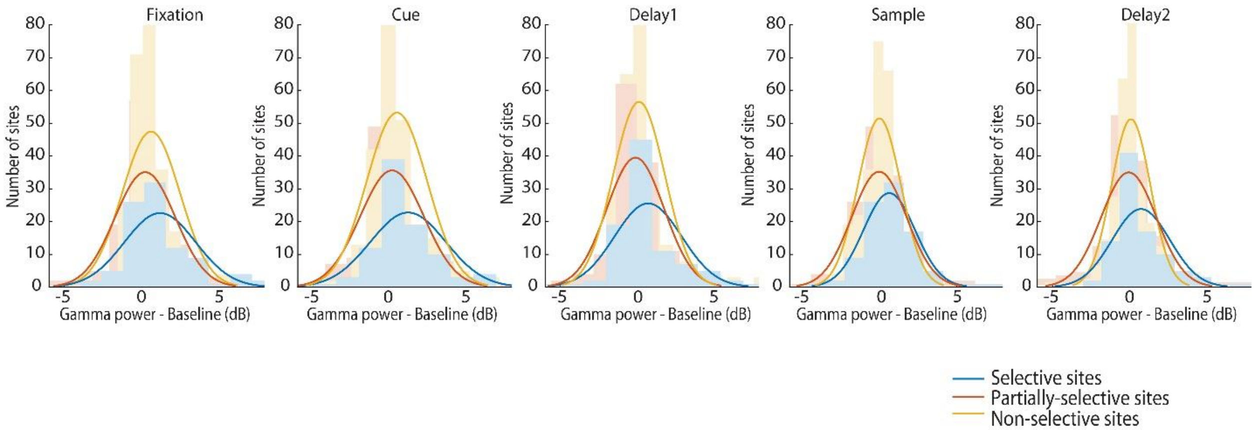

Supplementary Fig. 2: Distribution of gamma power across sites. LFP induced spectral power recorded with the spatial stimulus set from the prefrontal cortex, after training. Histograms of spectral power from selective, partially selective, and non-selective sites is shown separately in each task epoch (n=94, 133, 273 respectively). Continuous lines are Gaussian fits. Gamma power was calculated after subtracting power computed in the inter-trial interval in the same frequency range.

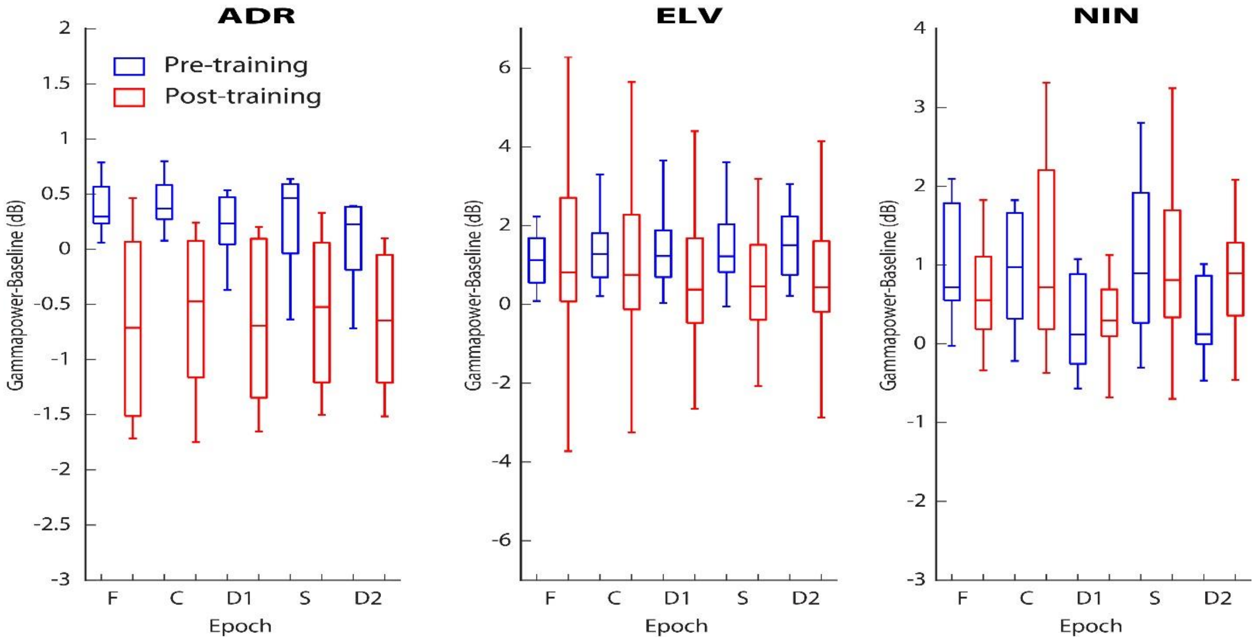

Supplementary Fig. 3: Gamma power changes across subjects. LFP induced spectral power recorded with the spatial stimulus set from the prefrontal cortex, before and after training. Spectral power from each monkey (ADR, ELV, NIN) is shown separately in each task epoch (Fixation-F, Cue-C, First Delay-D1, Sample-S, Second Delay-D2). Box plots represent the median power and first and third quartile; whiskers represent 1.5 times the interquartile range. Gamma power was calculated after subtracting power computed in the inter-trial interval in the same frequency range, in selective sites. Number of sites in ADR: n= 7, 6 for pre-training and post-training phases; ELV n=30,74, respectively; NIN n=7,14, respectively.

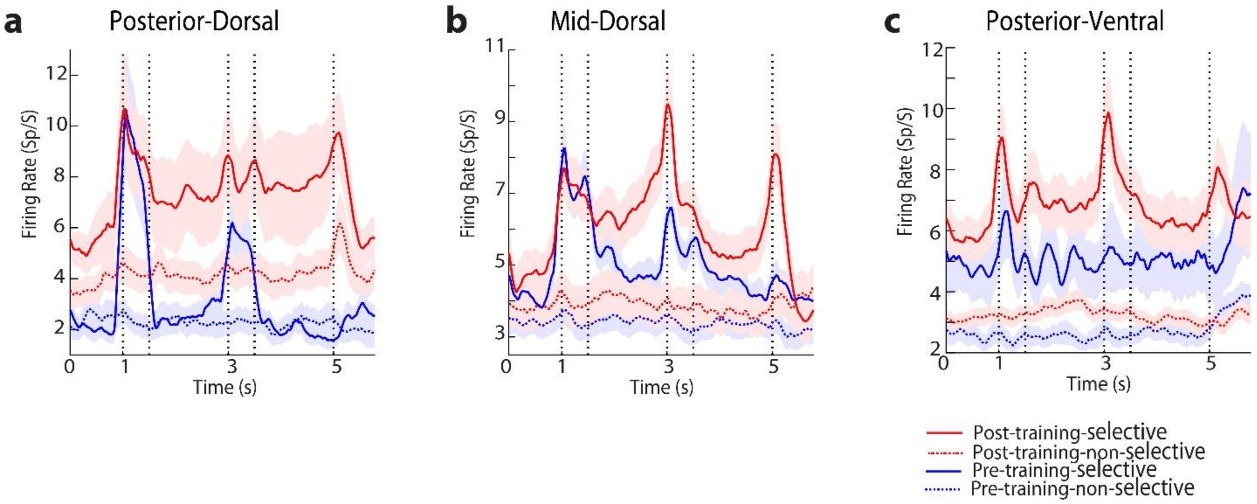

Supplementary Fig. 4: Firing rate in the spatial task, for each prefrontal subdivision, prior to training and after the training. **a** Population averages of neuronal firing rate from posterior-dorsal prefrontal cortex: the mean firing rate is shown separately for selective (N= 34, 64) and non-selective sites (N=50, 117) from prior to training and after training, respectively. **b** Mid-dorsal: mean firing rate is shown separately for selective (N= 172, 175) and non-selective sites (N=269, 186) from prior to training and after the training, respectively. **c** Posterior-ventral: mean firing rate is shown separately for selective (N= 36, 83) and non-selective sites (N=233, 334) from prior to training and after the training, respectively.

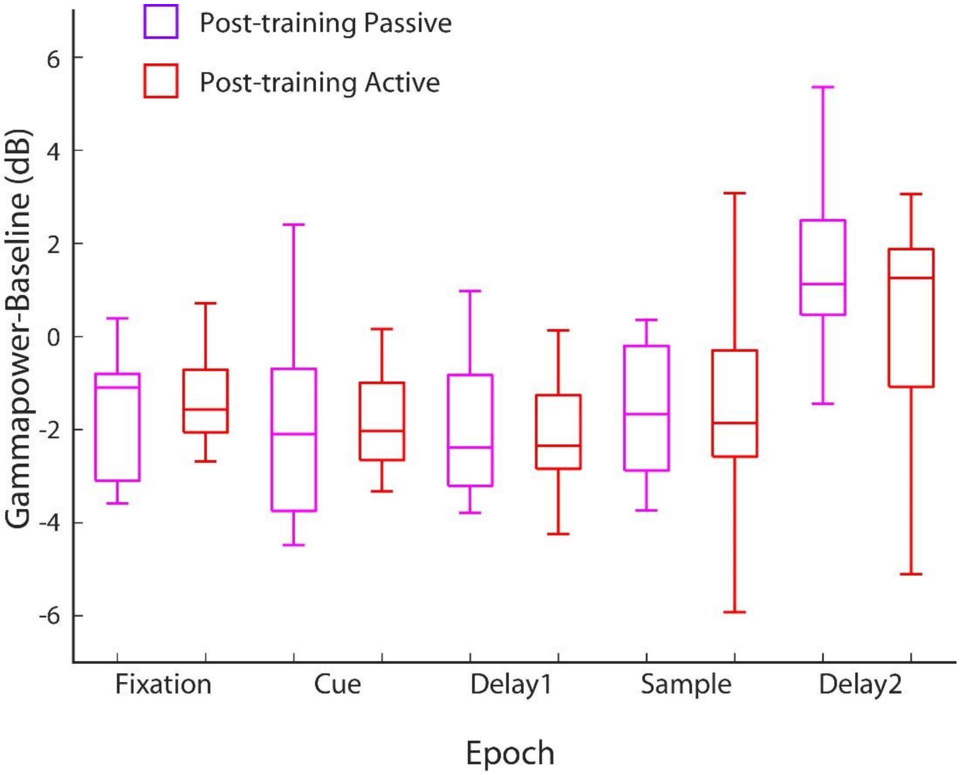

Supplementary Fig. 5: Gamma power in passive and active sessions after training. LFP induced spectral power recorded with the spatial stimulus set from the prefrontal cortex, after training, when subjects performed the task or viewed stimuli passively. Box plots represent the median power and first and third quartile; whiskers represent 1.5 times the interquartile range. Gamma power was calculated after subtracting power computed in the inter-trial interval in the same frequency range. The box plots show gamma power from selective sites tested with both the passive and active task (N=16).

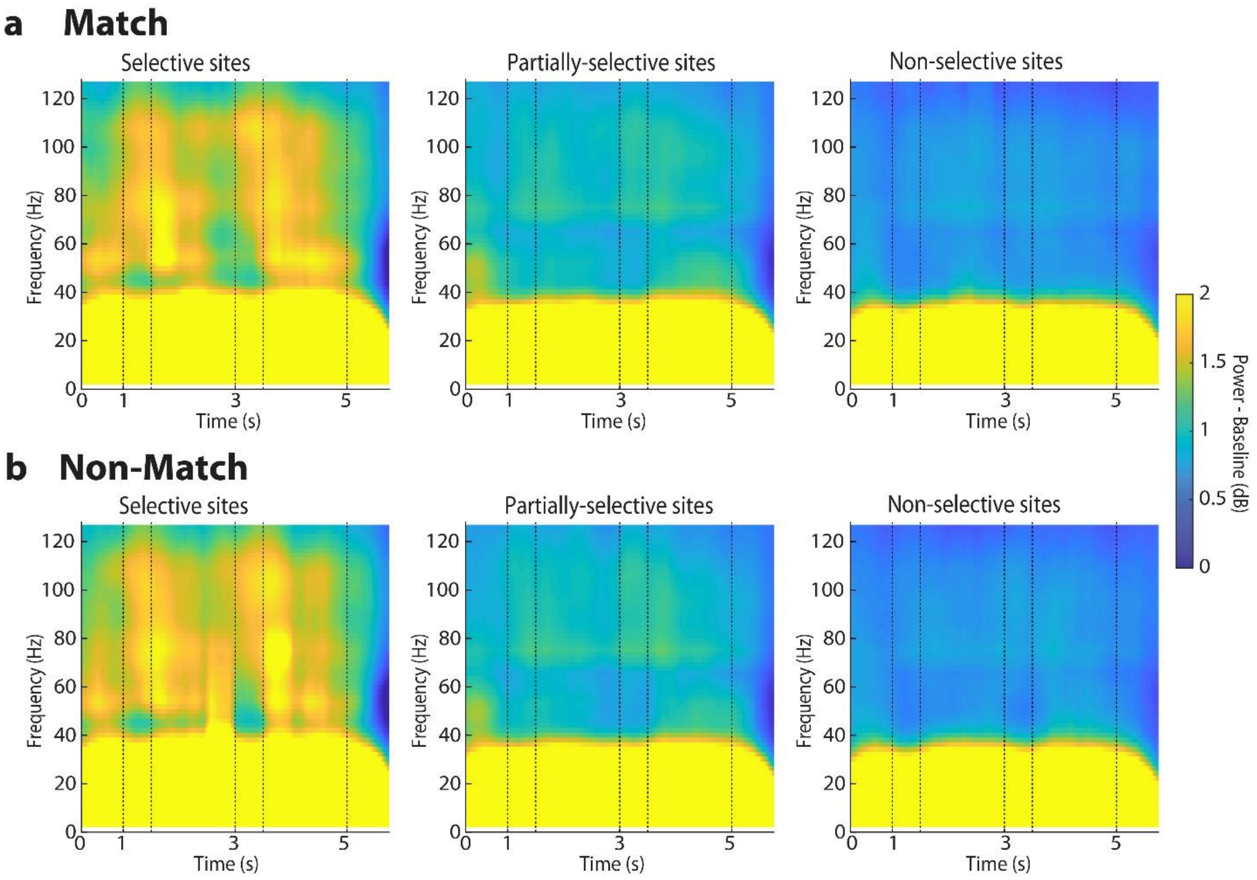

Supplementary Fig. 6: LFP spectral power for match and nonmatch stimuli prior to training in spatial task. LFP induced spectral power is plotted separately for target choice (Match/Non Match) recorded with the spatial stimulus set from the prefrontal cortex, prior to training. **a** Results are shown separately for match trials (n= 3445, 11090 and 13961 trials for selective, partially-selective, and non-selective sites respectively. **b** Results from non-match trials (n=3441, 11116 and 13988 trials for selective, partially-selective, and non-selective sites, respectively).

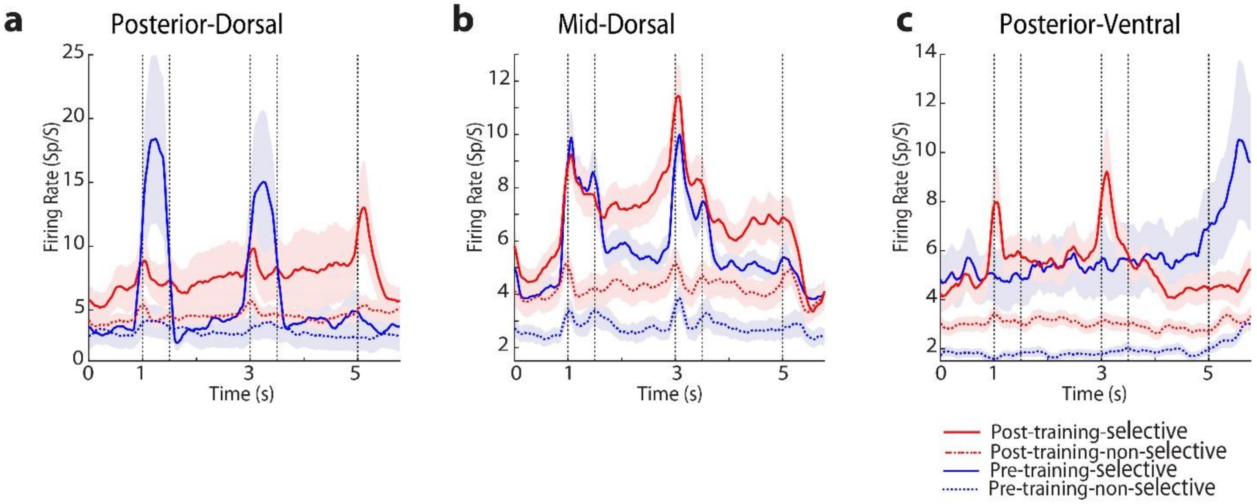

Supplementary Fig. 7: Firing rate in shape task, for each prefrontal subdivision. Neuronal spiking in the spatial stimulus set prior to and after training. Population averages of neuronal firing rate from each prefrontal subdivision. **a** Posterior-dorsal: mean firing rate is shown separately for selective (N= 14, 45) and non-selective sites (N=65, 130), prior to training and after training, respectively. **b** Mid-dorsal: mean firing rate for selective (N= 117, 131) and non-selective sites (N=166, 208), prior to training and after training, respectively. **c** Mean firing rate for selective (N= 36, 62) and non-selective sites (N=202, 287) prior to training and after the training, respectively.

Post-Training

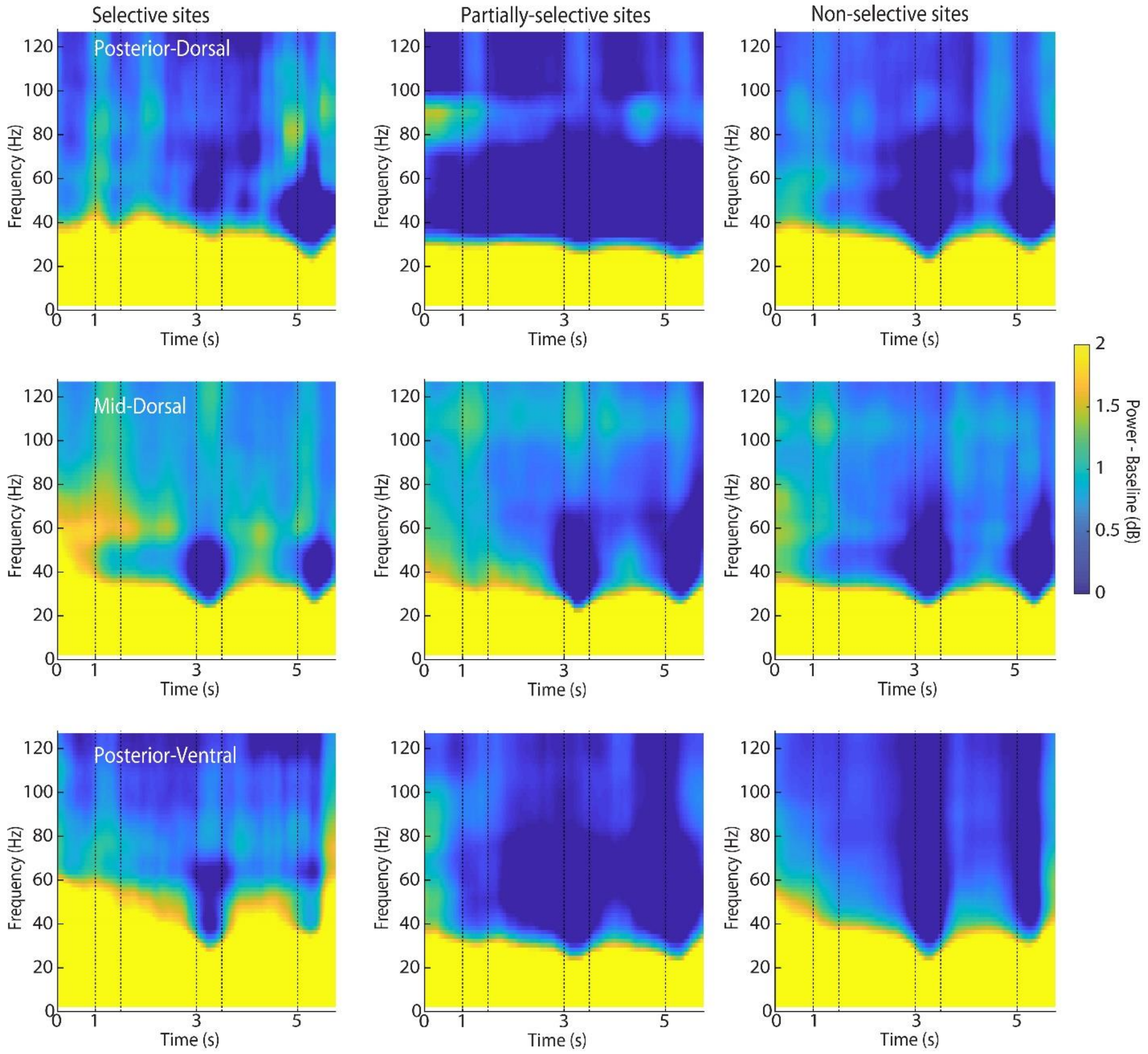

Supplementary Fig. 8: LFP spectral power for shape task in each prefrontal subdivision, after training. Mean spectral power is shown separately for the posterior-dorsal subregion of the prefrontal cortex (n=513, 2568, 3290 trials for selective, partially-selective sites, non-selective sites respectively); for the mid-dorsal region (n= 2946, 6214, 5831); and for the posterior-ventral subregion (n=824, 4450, 11113).

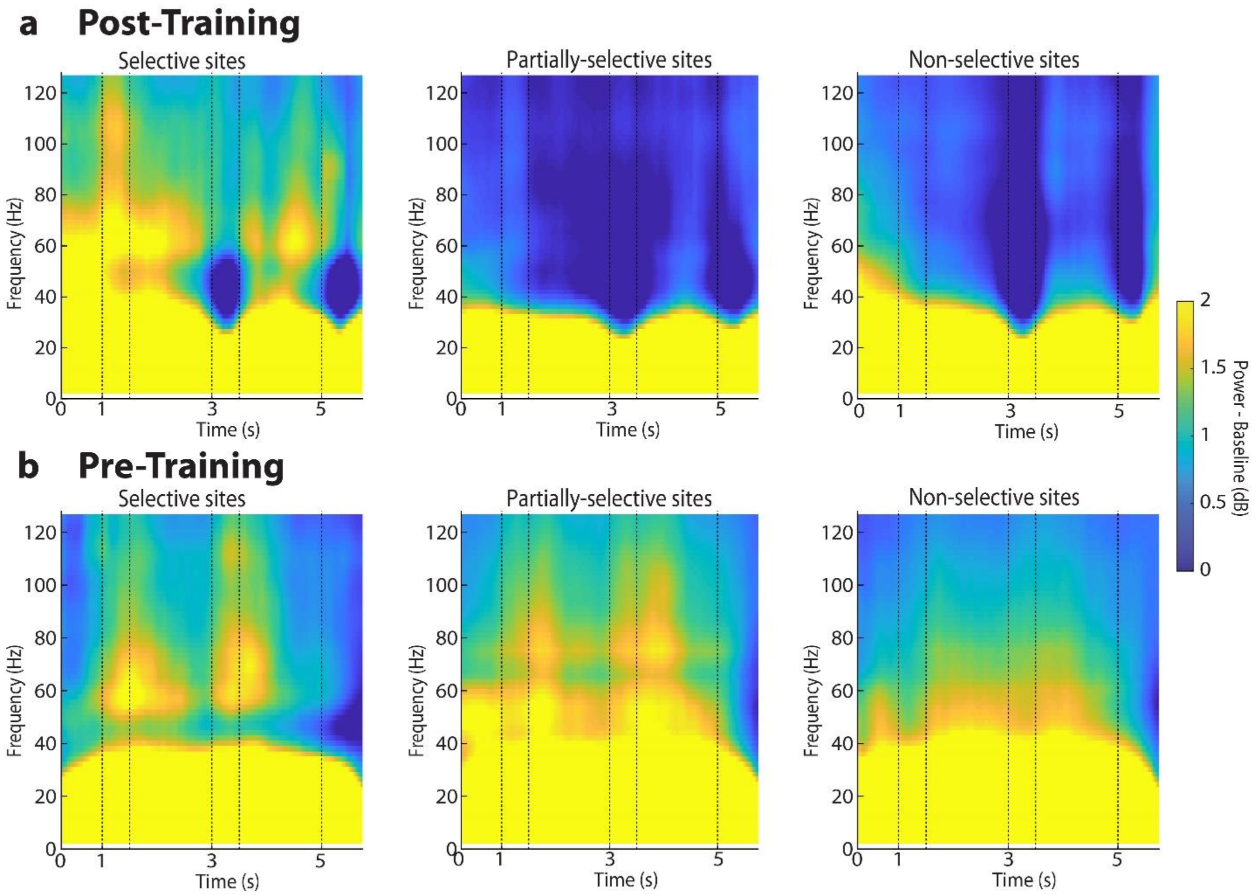

Supplementary Fig. 9: LFP spectral power for the shape working memory task, with sites sorted based on spatial selectivity. a Mean LFP induced spectral power recorded with the shape stimulus set from the prefrontal cortex, post training. Spectral power from sites that were selective in the spatial task (which was tested in a different block of trials). Results are shown separately from selective sites (n=2496 trials) in the left column, partially-selective sites (n=4260 trials) in the middle column and non-selective sites (n=12154 trials) in right column. **b** Similarly, results are shown separately from selective sites (n=1636), partially-selective sites (n=5341) and non-selective sites (n=8760) as in A, prior to training.
